# Chemistry, Meteorology, and the Function of Digestion Considered with Reference to Natural Theology

**Published:** 1846-04

**Authors:** 


Chemistry, Metereology, and the Function of Digestion, considered with reference to Natural Theology. By *W. Prout,* M.D., F.R.S. Third Edition. Octavo, pp. 510. London, 1845.

The fact of Dr. Prout's Bridgewater Treatise having reached its third edition proves it requires no recommendation on our parts to secure it an extensive perusal; and, in fact, so well known are now the views of its able author, that we only notice the present issue for the purpose of stating that he justly cites many of the results of the investigations of Liebig and other chemists as confirmations of the views of assimilation and nutrition promulgated by him many years since. We have indeed often thought of late, that, in the deserved admiration which has been bestowed upon the ingenious research and happy generalizations of the celebrated German chemist, we have in this country been somewhat ungratefully forgetful of the labours of Dr. Prout, and have received much as absolutely new, which was in fact but an old friend with a new face. Dr. Prout, after describing his classification of aliments into the saccharine, oily, and caseous elements, and the processes of transformation these undergo, observes—

"These views of aliments and of assimilation were first published by the author in their present form in 1834, and are now generally admitted by physiologists. They had been partially made known many years before the period stated, but attracted little notice in this country until they were adopted by MM. Liebig and Dumas, through whose means chiefly they have become generally known. MM. Liebig and Dumas have attempted to carry these views farther; they maintain that the chief or only use of saccharine and oleaginous elements is to produce animal heat by the combustion of the carbon they contain. We dissent from this opinion, for reasons, some of which will be subsequently noticed."

When treating of *Animal Heat,* he again adverts to this opinion.

"The quantity of carbon thrown off by the lungs, is very abundant; but has probably been much over-rated. Philosophers formerly estimated that the lungs of a man of ordinary size expel, in the course of 24 hours, about 11 ounces of carbon; but M. Liebig has recently estimated the quantity of carbon daily taken, by a man in his food, and expelled as carbonic acid gas, at no less than 13·9 ounces! This celebrated chemist, also, in adopting the views above given, respecting the source of animal heat, has pushed them much further, and maintains that animal heat is *entirely* the result of the union of the hydrogen and carbon in the system with oxygen; and that the chief use of non-azotized articles of food, *i. e.* of saccharine and oleaginous aliments, is to furnish carbon and hydrogen for combustion, and for the maintenance of animal heat! We have already said, and with many others always admitted, that the union of oxygen with hydrogen and carbon is *one* source of animal heat; but at present we totally dissent from the opinion that such union of oxygen with hydrogen and carbon is the *only* source of animal heat; or that the only use of saccharine and oleaginous aliments is that assigned.* The subject of Respiration and Animal Heat, notwithstanding the attention they have received from physiologists, are, we think, very imperfectly understood; and, if we are not mistaken, these important functions will hereafter be placed in an altogether different light from that in which they are at present contemplated."

There are other conclusions of the German chemists, which Dr. Prout does not accept. Of *Proteine* he thus remarks.

"An attempt has recently been made to shew that albumen, fibrin, and caseine contain a certain common fundamental proximate element, to which the name of *Proteine* has been given. That such a common proximate element may be derived from albumen, fibrin, and caseine, is not denied. Viewed indeed in connexion with organization and life, the supposition that some common proximate element adapted for ulterior changes exists in animal bodies is very probable; and accords well with the simplicity of natural operations. But that a substance, obtained like proteine by the rude and disorganizing processes of common chemistry, should be that common proximate element; or that such a substance should ever be employed at all in vital operations without undergoing the preliminary assimilating processes, is more than at present we are disposed to admit."

* "We had intended to state some objections to these assumptions, but have thought it better to refer the reader to a treatise on the Statics of the Human Chest, Animal Heat, &c., by Julius Jeffreys, Esq., where some arguments will be found against them, which appear to us unanswerable."

